# Thermally Stable and Reusable Ceramic Encapsulated and Cross-Linked CalB Enzyme Particles for Rapid Hydrolysis and Esterification

**DOI:** 10.3390/ijms23052459

**Published:** 2022-02-23

**Authors:** Min Song, Jeong-Ho Chang

**Affiliations:** 1 Korea Institute of Ceramic Engineering and Technology, Cheongju 28160, Korea; sm_0704@naver.com; 2 Department of Materials Science & Engineering, Yonsei University, Seoul 03722, Korea

**Keywords:** thermal stability, reusability, silica, encapsulation, cross-linking, CalB enzyme, benzyl benzoate

## Abstract

*Candida antarctica* lipase B (*CalB*) enzyme was encapsulated and cross-linked by silica matrix to enhance its thermal stability and reusability, and demonstrated an enzymatic ability for rapid hydrolysis and esterification. Silica encapsulated CalB particles (Si-E-CPs) and silica cross-linked CalB particles (Si-CL-CPs) were prepared as a function of TEOS concentration. The particle size analysis, thermal stability, catalytic activity in different pHs, and reusability of Si-E-CPs and Si-CL-CPs were demonstrated. Furthermore, the determination of the CalB enzyme in Si-E-CPs and Si-CL-CPs was achieved by Bradford assay and TGA analysis. Enzymatic hydrolysis was performed against the *p*-nitrophenyl butyrate and the catalytic parameters (K_m_, V_max_, and K_cat_) were calculated by the Michaelis–Menten equation and a Lineweaver–Burk plot. Moreover, enzymatic synthesis for benzyl benzoate was demonstrated by esterification with an acyl donor of benzoic acid and two acyl donors of benzoic anhydride. Although the conversion efficiency of Si-CL-CPs was not much higher than that of native CalB, it has an efficiency of 91% compared to native CalB and is expected to be very useful because it has high thermal and pH stability and excellent reusability.

## 1. Introduction

Lipases have been used in various industries, such as agriculture, medicine, pharmaceuticals, and cosmetics, due to their high activity and regioselectivity [1,2,3,4,5]. Lipases work under mild conditions (neutral pH, low temperature) and are completely biodegradable, to reduce chemical consumption and waste generation, attracting attention as a green biocatalyst [6,7,8]. Lipases generally catalyze a hydrolysis reaction, but they effectively perform synthesis reactions (esterification, acylation, etc.) in a non-aqueous or low-water environment [9,10]. Among lipases, *Candida antarctica* lipase B (CalB) is the prominent biocatalyst in several industrial applications, due to its wide range of substrates, high efficiency and enantioselectivity, thermal stability, and stability in organic solvents [11,12,13]. Structurally, CalB is composed of catalytic triad (Ser, His, and Asp/Glu) within an acyl binding pocket [14]. The active site consists of the acyl binding pocket and a binding pocket for the moiety of secondary alcohols [11]. Furthermore, the active site of CalB is not covered by a lid and, thus, the active site for the hydrophobic substrate is directly accessible to the solvent [15]. CalB is used in a wide range of organic syntheses, such as aminolysis, esterification, and transesterification, due to those characteristics [16].

However, lipases suffer several drawbacks in the commercialization process. Lipases are difficult to recover from an aqueous reaction mixture due to their high solubility in water [17]. Also, it is difficult to maintain the structural stability of proteins during biochemical reactions, which may affect the biological activity of lipase [18,19]. Generally, lipases are sensitive to process conditions other than the optimal conditions, normally a narrow pH range, and low thermal stability [15]. In addition, lipases incur high costs during production and processing due to their low long-term operation stability and short storage life [20,21].

Enzyme immobilization is an efficient method that can overcome these limitations. Enzyme immobilization improves the stability of lipase and allows easy recovery from an aqueous reaction mixture, reuse of lipase, and a continuous operation process [22,23]. The cost of using encapsulated lipase in continuous processes is more than 20 times lower than that of conventional processes, which can effectively reduce costs [19,23,24]. In general, the enzyme immobilization method can be divided into binding or encapsulating to an organic and inorganic support, and by cross-linking of the protein molecule [25,26]. Binding to a carrier can enhance lipase stability by preventing direct contact with the external environment and improve resistance to thermal and chemical denaturation [27,28,29,30]. Enzyme encapsulation technology using a silica matrix has recently been demonstrated as an effective method for enzyme entrapment [31,32]. A silica matrix is chemically inert and has higher mechanical strength than most organic polymers and, thus, works stably in organic synthesis [33,34]. Moreover, silica gels can easily be tailored to network textures, a large range of porous surfaces and processing conditions [33]. However, they may produce low productivity and effects on enzymatic activity due to lipase distortion between the lipase and the support, active site blocking by the silica support and diffusion problems [35,36]. In contrast, immobilization by the cross-linking of enzyme molecules, which we called cross-linked enzyme aggregates (CLEA), is a carrier-free method and comprises active enzymes [37,38]. Thus, the volumetric activity of CLEA is much higher than that of both conventional and immobilized enzymes [39]. However, CLEA exhibited low activity retention, and low stability in mechanical strength and under heat and organic solvents [25]. Therefore, it is necessary to study immobilized enzymes that are stable from protein denaturation, while having high enzymatic activity.

**Figure 1:**
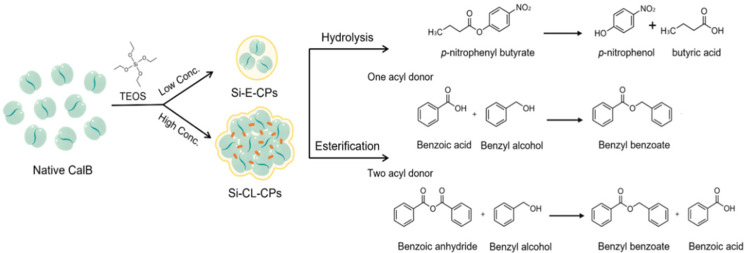
Schemes of silica encapsulation and cross-linking on CalB enzyme with different.

In this work, *Candida antarctica* lipase B (CalB) was encapsulated and cross-linked by tetraethyl orthosilicate (TEOS), as a silica precursor to enhance the enzyme stability. Silica-encapsulated CalB particles (Si-E-CPs) and silica-cross-linked CalB particles (Si-CL-CPs) were prepared as a function of TEOS concentration, in a range of 10 mM to 300 mM. The particle size analysis, thermal stability (25–65 °C), catalytic activity in different pHs (pH 5–9), and reuseability of Si-E-CPs and Si-CL-CPs were demonstrated in comparison a with native CalB enzyme. Furthermore, the determination of the CalB enzyme in Si-E-CPs and Si-CL-CPs was achieved by Bradford assay and TGA analysis. With these materials, we achieved enzymatic hydrolysis and a synthesis reaction, as shown in Figure 1. Enzymatic hydrolysis was performed against the *p*-nitrophenyl butyrate (*p*-NPB) and the catalytic parameters, such as K_m_, V_max_, and K_cat,_ which were calculated by the Michaelis–Menten equation and Lineweaver–Burk plot.

TEOS concentrations and application to enzymatic hydrolysis and esterification.

In addition, we synthesized benzyl benzoate, an aromatic ester, by esterification and acylation using two acyl donors, benzoic anhydride and benzoic acid with native CalB and Si-CL-CPs. The synthetic efficiency for benzyl benzoate with native CalB and Si-CL-CPs was calculated and an available mechanism is suggested. 

## 2. Results and Discussion

### 2.1. Preparation and Characterization of Si-E-CPs and Si-CL-CPs

**Figure 2:**
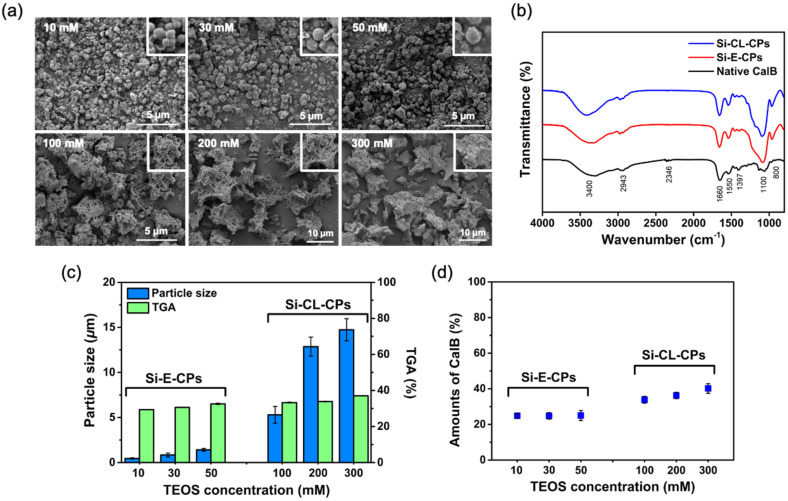
The (**a**) SEM images of silica encapsulation and cross-linking on CalB enzyme as a function of TEOS concentration, (**b**) FT-IR spectra of native CalB, Si-E-CPs, and Si-CL-CPs), (**c**) correlations of particle sizes and TGA analysis of Si-E-CPs and Si-CL-CPs as a function of TEOS concentration, and (**d**) determination of CalB enzyme in Si-E-CPs and Si-CL-CPs by Bradford assay.

Si-E-CPs and Si-CL-CPs were prepared by control of the TEOS concentration on the CalB enzyme, in the range of 10 to 50 mM, and 100 to 300 mM, respectively. Figure 2a shows the SEM images of the prepared Si-E-CPs, and Si-CL-CPs with different particles sizes by TEOS concentrations. At lower concentrations, such as 10 mM, 30 mM, and 50 mM of TEOS on the CalB enzyme, Si-E-CPs were uniformly spherical particles and showed the size of 0.46 μm, 0.89 μm, and 1.41 μm, respectively. At higher concentration of TEOS, such as 100 mM, 200 mM, and 300 mM, Si-CL-CPs were obtained with increasing particle sizes of 5.30 µm, 12.86 µm, and 14.73 µm, respectively. In the sol-gel reaction, when the concentration of TEOS increases and reaches the supersaturation region, both the hydrolysis and condensation rates are accelerated, increasing the consumption rate of the intermediate through the condensation reaction. This phenomenon causes aggregation of the silica and the enzyme, and the generated gel networks act as a cross-linking agent between the enzyme and the silica. Therefore, the particle size of synthesized silica-enzyme aggregates are relatively larger than colloidal silica [40,41,42]. Fourier transform infrared (FT-IR) spectra of native CalB, Si-E-CPs, and Si-CL-CPs are shown in Figure 2b. The several characteristic vibrations for each sample were observed as follows. The N-H stretching and O-H stretching vibrations were observed at 1660 cm^−1^ and 1550 cm^−1^ and at 3400 cm^−1^, respectively. Moreover, the asymmetric and symmetric stretching vibrations of Si–O–Si bond are seen at 1100 cm^−1^ and 800 cm^−1^, in which this result indicated that the native CalB enzyme was successfully encapsulated and cross-linked with silica precursor TEOS. 

Figure 2c shows the relationship between the particle size and thermogravimetric analysis (TGA) of Si-E-CPs and Si-CL-CPs as a function of the TEOS concentration. TGA analysis was performed to investigate the amount of CalB enzyme in Si-E-CPs and Si-CL-CPs and was calculated in the temperature range of 200–500 °C. At lower concentrations such as 10 mM, 30 mM, and 50 mM of TEOS, the amounts of CalB enzyme in Si-E-CPs were slightly increased to 29.78%, 31.86% and 33.13%, respectively. At higher concentrations of TEOS such as 100 mM, 200 mM, and 300 mM, the amounts of CalB enzyme in Si-CL-CPs were 33.96%, 34.28%, and 37.08%, respectively. The amount of CalB enzyme in Si-E-CPs and Si-CL-CPs particles seems to increase slightly with increasing reactive TEOS concentration, but the amount of CalB enzyme reacting with silica is always constant and therefore the same. In addition, Bradford assay was further demonstrated using Coomassie brilliant blue reagent to determine the CalB enzyme in Si-E-CPs and Si-CL-CPs as a function of TEOS concentration as shown in Figure 2d. The amounts of CalB enzyme as a function of TEOS concentration were 24.83%, 24.80%, 24.97%, corresponding to 10 mM, 30 mM, and 50 mM, and 33.83%, 36. 22% and 40.17% corresponding to 100 mM, 200 mM, and 300 mM, respectively. The average amounts of CalB enzymes in Si-E-CPs and Si-CL-CPs were 24.87% and 36.74%, respectively. The concentration of TEOS increased in Si-E-CPs, but the amount of CalB enzyme in Si-E-CPs was almost the same as the amount of CalB enzyme reacting with silica, which was constant. In addition, the amount of CalB enzyme in Si-CL-CPs was 36.74%, which was higher than that of Si-E-CPs because the high concentration of TEOS randomly contained the CalB enzyme remaining around silica during a very fast sol-gel condensation reaction.

### 2.2. Thermal Stability, pH Activity, and Reusability of Si-E-CPs and Si-CL-CPs

**Figure 3:**
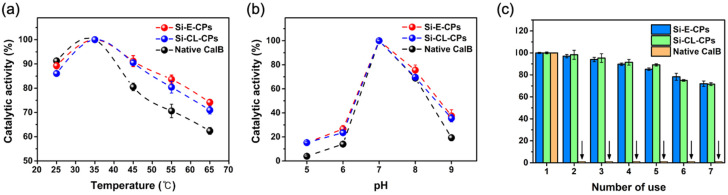
(**a**) Thermal stability and (**b**) catalytic activity in pHs with native CalB, Si-E-CPs, and Si-CL-CPs, and (**c**) reusability of Si-E-CPs and Si-CL-CPs.

Determination of thermal stability and pH activity for native CalB, Si-E-CPs, and Si-CL-CPs are very important to understand how lipase encapsulation affects enzyme activity. In general, sol-gel encapsulated enzymes have been reported to have higher stability than native CalB [43]. The catalytic activities of native CalB, Si-E-CPs, and Si-CL-CPs were investigated with hydrolysis of p-NPB, in the temperature range of 25–65 °C to access the thermal stability of the enzyme. Figure 3a shows the catalytic activity of native CalB, Si-E-CPs, and Si-CL-CPs as a function of temperature. The optimum thermal activities are seen at 35 °C, and the activities decreased at higher temperatures, above 35 °C. Moreover, Si-E-CPs and Si-CL-CPs are much more stable than the native CalB due to the encapsulated and cross-linked silica matrix. The thermal stability of Si-E-CPs is slightly better than that of Si-CL-CPs by the perfect silica encapsulation. Figure 3b shows the effect of pH on the catalytic activity of native CalB, Si-E-CPs, and Si-CL-CPs, in the pH range of 5–9. The optimum pH for catalytic activities of the samples is seen at pH 7 and the activities decreased at lower and higher pH. This result means that the activity of the enzyme, according to the pH change, depends on the catalytic activity at a specific pH of the enzyme, regardless of the silica immobilization process, such as encapsulation and cross-linking. Figure 3c shows the comparison of reusability with Si-E-CPs, and Si-CL-CPs. The greatest advantage of silica immobilization on the enzyme is that expensive enzymes can be repeatedly used. The native CalB enzyme is easily dissolved in an aqueous solution and hard to re-use because the recovery is not easy. To evaluate the reusability, Si-E-CPs and Si-CL-CPs were recovered seven times and the Si-E-CPs and Si-CL-CPs were washed with buffer solution after each cycle.

As a result, it was confirmed that the catalytic activity of Si-E-CPs and Si-CL-CPs was still maintained over 90% after fifth usage. These results mean that the Si-E-CPs and Si-CL-CPs can be used efficiently in a continuous process without activity loss.

### 2.3. Enzymatic Hydrolysis for p-Nitrophenyl Butyrate

**Figure 4:**
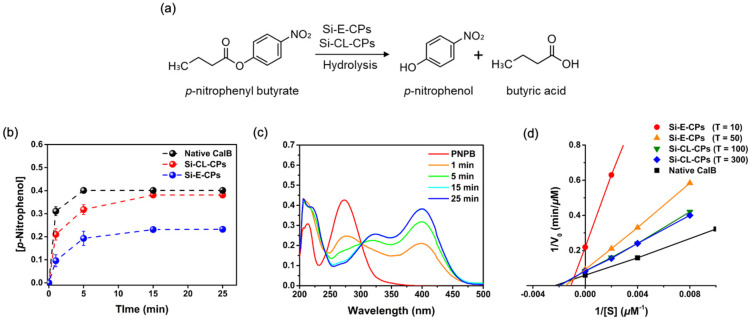
(**a**) Schemes of enzymatic hydrolysis with Si-CL-CPs against p-NPB to p-NP with native CalB, Si-E-CPs, and Si-CL-CPs (**b**) absorption spectra of p-NPB as a function of reaction time, and (**c**) concentration of formed p-NP during the enzymatic hydrolysis with native CalB, Si-E-CPs, and Si-CL-CPs, and (**d**) Lineweaver–Burk plots and Michaelis–Menten parameters of native CalB, Si-E-CPs and Si-CL-CPs as a function of TEOS concentration, respectively.

Enzymatic hydrolysis was demonstrated for the hydrolysis of p-nitrophenyl butyrate (p-NPB), using native CalB, Si-E-CPs, and Si-CL-CPs, as shown in Figure 4a. The p-NPB is hydrolyzed to p-nitrophenol (p-NP) and butyric acid by enzyme catalysts. The absorbance of the product p-NP was measured with a UV-Vis. spectrophotometer at a wavelength of 400 nm. Figure 4b shows the concentration of the formed p-NP through the hydrolysis of p-NPB for native CalB, Si-E-CPs, and Si-CL-CPs, respectively. The enzymatic p-NPB hydrolysis in native CalB, Si-E-CPs, and Si-CL-CPs was shown in the order of native CalB > Si-CL-CPs > Si-E-CPs at the initial reaction. This is due to several reasons. First, the higher the amount of CalB contained in the catalyst, the faster the catalytic reaction. Calculating the amount of CalB in native CalB, Si-E-CPs, Si-CL-CPs for enzymatic p-NPB hydrolysis is 36.79 μg, 36.74 μg, 24.87 μg, respectively, since 24.87% and 36.74% of CalB are contained in the Si-E-CPs, and Si-CL-CPs on average, the amount of CalB in 0.1 mg of each Si-E-CPs, Si-CL-CPs is calculated. The production efficiency of p-NP is shown in the order of native CalB > Si-CL-CPs > Si-E-CPs, which has a tendency of CalB > Si-CL-CPs > Si-E-CPs, such as the order of CalB content of the catalyst used. It is noteworthy that, although the amount of native CalB and the amount of CalB in Si-CL-CPs are almost the same as 36.79 μg and 36.74 μg, the efficiency of p-NP production is much better in native CalB than in Si-CL-CPs. This shows that the active sites of CalB enzyme are masked or shielded by bonding with silica, so that the hydrolysis efficiency of Si-CL-CPs is lower than that of native CalB. Second, when comparing the silica cross-linking process with the encapsulation process, the active sites of the CalB enzyme that can interact with the p-NPB substrate are masked or shielded more in the encapsulation process than in the cross-linking process, so that the hydrolysis efficiency is improved, such as Si-CL-CPs > Si-E-CPs. Third, the amount of CalB of Si-E-CPs by the encapsulation process is 24.87 μg, which is less than 36.74 μg of Si-CL-CPs, so it can be inferred that the efficiency of p-NP production is lower than that of Si-CL-CPs. In addition, considering the internal diffusion limitations of Si-E-CPs and Si-CL-CPs, it is expected that Si-CL-CP will be much smaller than that of Si-E-CP. The internal diffusion limitation depends on the pores and particle size of the catalyst, and the pore and particle size of Si-CL-CP are 23.5 nm and 12.8 μm, which is larger than the 9.1 nm and 1.4 μm of Si-E-CPs. Therefore, since the internal diffusion limit of Si-CL-CPs is relatively smaller than that of Si-E-CPs, the formation efficiency of p-NPs is relatively better. Figure 4c shows the absorption spectra of p-NPB as a function of the reaction time with Si-CL-CPs. As the reaction time increased, the concentration of formed p-NP increased and was seen at 400 nm. Moreover, the reactant, p-NPB gradually disappeared according to the reaction time. After 25 min, it was completely hydrolyzed, and no absorbance band was observed at 270 nm. Consequently, an isosbestic point was observed at 300 nm. 

An isosbestic point is a wavelength at which two chromophores possess the same extinction coefficient. Therefore, this means that two absorbing components, such as p-NPB and p-NP, are in equilibrium and their relative proportions are controlled by the concentration of another component. At wavelengths before 300 nm, p-NPB, a reactant, was present in the solution, and as the reaction of silica-CalB catalysts proceeded, it means that p-NP was formed at wavelengths after 300 nm. Figure 4d shows the enzyme kinetics of the p-NP with native CalB, Si-E-CPs, and Si-CL-CPs from Lineweaver–Burk plots and Michaelis–Menten kinetics. Various enzyme kinetic parameters were calculated from Lineweaver–Burk plots. The Michaelis–Menten constant (K_m_) shows affinity between enzymes and substrates, and the lower the value, the better the enzyme binds to the substrate [44]. The turnover number (K_cat_) refers to the number of times each enzyme site converts substrate to product per unit time and the K_cat_/K_m_ ratio has been used as a measure of enzyme performance. The enzyme kinetic parameters, such as K_m_, K_cat_ and V_max_ against native CalB, Si-E-CPs, and Si-CL-CPs are shown in Table 1. The K_m_ value of native CalB was 432.60 μM, which is lower than the values of Si-E-CPs and Si-CL-CPs, and as the concentration of TEOS increased to 10 mM, 50 mM, 100.0 mM, and 300 mM, the K_m_ values decreased to 915.32 μM, 588.31 μM, 517.31 μM, and 497.86 μM, respectively. However, the V_max_ value of native CalB was 16.86 μMmin^−1^, which decreased to 4.57 μMmin^−1^, 9.94 μMmin^−1^, 12.02 μMmin^−1^, and 12.06 μMmin^−1^, as the concentration of TEOS increased. The value of V_max_ is almost constant at concentrations greater than 100 mM. The K_cat_ values of native CalB, Si-E-CPs and Si-CL-CPs show a similar tendency to V_max_. In Si-E-CPs and Si-CL-CPs, the K_cat_ values increased to 5.03 min^−1^, 9.90 min^−1^, 10.70 min^−1^, and 10.73 min^−1^ as the concentration of TEOS increased. Consequently, the catalytic efficiency (K_cat_/K_m_) shows the order of native CalB > Si-CL-CPs >Si-E-CPs, where the efficiency of Si-E-CPs and Si-CL-CPs is slightly lower than that of native CalB, but it has the advantage of convenient catalytic recovery and shortening time in the reaction process.

### 2.4. Enzymatic Esterification of Benzyl Benzoate with Si-CL-CPs

The lipase-catalyzed esterification reactions have gained increasing attention in many applications, due to an increased use of organic esters in biotechnology and the chemical industry [45]. For this reason, esterification by lipases has been employed in experiments using either primary or secondary alcohols, or both, free-solvent systems, or organic solvents [46]. However, there are many difficulties in quantitative analysis using UV-Vis. and HPLC analysis because aliphatic esters obtained from primary or secondary alcohol do not respond to UV-Vis. spectroscopy. Therefore, for the qualitative analysis of aliphatic esters, generally, NMR spectroscopy is used, after separation and purification through column chromatography [47]. To facilitate the quantitative analysis using UV-Vis. spectroscopy of ester compounds, obtained from lipase-catalyzed esterification, lipase-catalyzed esterification was used to synthesize the aromatic ester compounds that are sensitive to UV-Vis. spectroscopy. 

**Figure 5:**
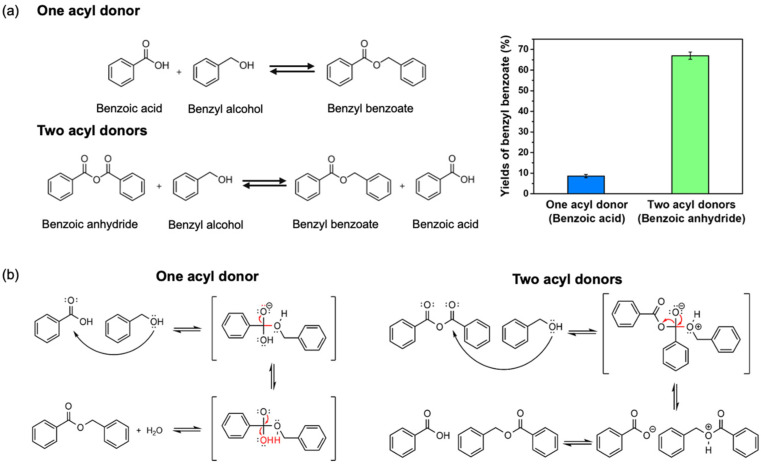
(**a**) Schemes of enzymatic esterification of benzyl benzoate with the different acyl donors (inserted is the comparison of yields of benzyl benzoate from benzoic anhydride and benzoic acid with Si-CL-CPs), and (**b**) suggested mechanism.

Typically, in this work, Si-CL-CPs were demonstrated for the lipase-catalyzed esterification of benzyl benzoate from one or two acyl donors. As shown in Figure 5a, the lipase-catalyzed esterification of benzyl benzoate with Si-CL-CPs is a result of the benzoic acid as an acyl donor and benzoic anhydride as two acyl donors in benzyl alcohol, respectively. Moreover, the yields of benzyl benzoate with Si-CL-CPs are 10% and 67%, corresponding to the esterification from benzoic acid (one acyl donor) and benzoic anhydride (two acyl donors). This is attributed to benzoic anhydride having two acyl donors (the first acyl donor forms benzyl benzoate and the second acyl donor forms benzoic acid). At this time, the formed benzoic acid may react with residual alcohol and lead to a secondary ester reaction, as shown in Figure 5b [48]. These results indicate that benzoic anhydride is suitable for benzyl benzoate synthesis. 

### 2.5. Conversion of Benzyl Benzoate from Benzoic Anhydride with Native CalB and Si-CL-CPs

**Figure 6:**
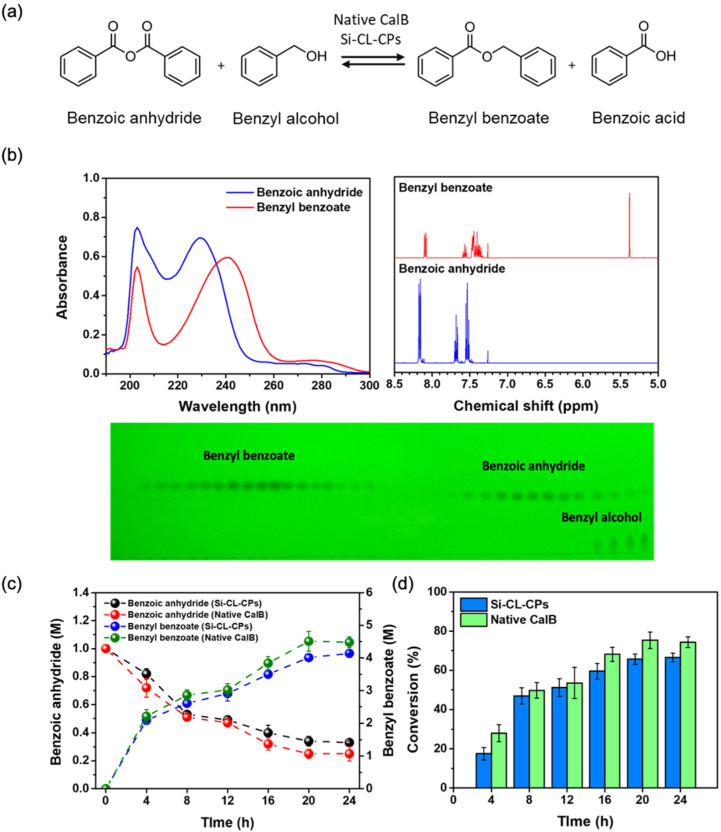
(**a**) Schemes of enzymatic esterification of benzyl benzoate with benzoic anhydride, (**b**) comparison of adsorption spectra and 1H-NMR spectra of benzoic anhydride and benzyl benzoate before and after reaction (inserted in the TLC image), (**c**) correlation of benzoic anhydride and benzyl benzoate concentration changes with native CalB and Si-CL-CPs, and (**d**) conversion efficiency of benzyl benzoate esterification with native CalB and Si-CL-CPs as a function of reaction.

As described above, in the lipase-catalyzed esterification of benzyl benzoates, benzoic anhydride having two acyl donors was superior to benzoic acid having one acyl donor. Therefore, the conversion efficiency of benzyl benzoate was evaluated using a native CalB enzyme and Si-CL-CPs, as shown in Figure 6a. Figure 6b shows the absorption spectra, proton nuclear magnetic resonance (^1^H-NMR) spectra, and thin layer chromatograph (TLC) image of benzoic anhydride and benzyl benzoate, separated by column chromatography, in which benzoic anhydride and benzyl benzoate show the maximum excitation, at 229 nm and 240 nm, respectively. The ^1^H-NMR spectra show chemical shifts of some characteristic peaks of benzoic anhydride and benzyl benzoate, as follows: 8.14–8.20 ppm (d, 4H), 7.65–7.71 ppm (t, 2H), and 7.51–7.57 ppm (t, 4H) for benzoic anhydride, and 8.07–8.12 ppm (d, 2H), 7.54–7.60 ppm (m, 1H), 7.28–7.49 ppm (m, 7H), 5.4 ppm (s, 2H) for benzyl benzoate, respectively. Moreover, the TLC image shows the perfect separation between benzyl benzoate and benzoic anhydride with the hexane: ethyl acetate (10:1) eluent. Figure 6c shows the change in concentration of benzoic anhydride and benzyl benzoate during the reaction time with native CalB and Si-CL-CPs. The lipase-catalyzed esterification of benzyl benzoates was carried out with a 1:9 molar ratio of benzoic anhydride and benzyl alcohol because an excessive alcohol may lead to secondary esterification with the acid [44,45,46]. During the reaction time for 24 h, the concentration of the benzyl benzoate product through the esterification is gradually increased to 4.51 M and 4.14 M, corresponding to native CalB and Si-CL-CPs, respectively. Moreover, the reactant benzoic anhydride decreased to 0.25 M and 0.33 M from 1 M, corresponding to native CalB and Si-CL-CPs, respectively. Figure 6d shows the conversion efficiency from benzoic anhydride to benzyl benzoate with native CalB and Si-CL-CPs. The *conversion* efficiency was calculated according to the following equation:

$$conversion\;\left(\%\right)=\frac{C_{i}-C_{f}}{C_{i}}\times 100$$

where *C_i_* and *C_f_* are the initial and final concentration of reactants (benzoic acid and benzoic anhydride), before and after enzymatic synthesis, respectively. Both native CalB and Si-CL-CPs showed the highest conversion efficiency at 24 h. The conversion efficiency was 75% and 67%, corresponding to native CalB and Si-CL-CPs, respectively. Although the conversion efficiency of Si-CL-CPs was not much higher than that of native CalB, it has an efficiency of 91%, compared to native CalB, and is expected to be very useful because it has high thermal and pH stability and excellent reusability.

## 3. Materials and Methods

### 3.1. Materials

Lipase B from Candida antarctica was obtained from Amicogen (33 kDa, Jinju, Korea) and used in encapsulation. Bovine serum albumin (BSA), Coomassie brilliant blue reagent, p-nitrophenyl butyrate (≥98%, p-NPB), tetraethyl orthosilicate (99%, TEOS), benzoic anhydride, benzoic acid, benzyl alcohol, trizma base, phosphate buffer saline (PBS) and sodium citrate were purchased from Sigma-Aldrich (Seoul, Korea). Hexane, ethyl acetate and hydrochloric acid were provided by Daejung (Seoul, Korea) and other chemicals were of analytical grade.

### 3.2. Preparation of Si-E-CPs and Si-CL-CPs

Si-E-CPs and Si-CL-CPs were prepared according to the sol-gel method [49,50,51]. Various concentrations (range, 11.5–307.2 mM) of TEOS and 1 mL of 0.1 M HCl were added in 10 mL of deionized water. To make a homogeneous solution, the mixed solution was vigorously agitated for 10 min at room temperature. Subsequently, 1.4 g of CalB lipase was suspended in 50 mL of phosphate buffer (PBS, 10 mM, pH 7.2). The lipase solution was added into the silica precursor solution. The mixture was intensively agitated for 15 min (gelation occurred after a few seconds) and continuously stirred for 8 h at 400 rpm. The obtained gel was freeze-dried for a day.

### 3.3. Determination of CalB Enzyme Amounts in Si-E-CPs and Si-CL-CPs

The amount of entrapped CalB enzyme was determined by the Bradford method using Coomassie brilliant blue reagent [52,53]. The CalB enzyme content was estimated comparing the concentration differences between the originally added enzyme solution and the washing solution. Bovine serum albumin (BSA) was used as a standard. The absorbance was measured at 595 nm by UV-Vis. spectrophotometry.

### 3.4. Thermal Stability and pH Activity of Native CalB, Si-E-CPs, and Si-CL-CPs

The thermal stability and the pH activity of native CalB, Si-E-CPs, and Si-CL-CPs were determined by the catalytic activity using p-NPB under various temperature (25–65 °C) and pH (0.1 M sodium citrate for pH 5–6, 0.1 M tris-HCl for pH 7–9) conditions. Each mixture was incubated for 20 min and centrifuged at 15,000 rpm for 1 min. Catalytic activities were calculated at optimum temperature and pH conditions.

### 3.5. Reusability of Si-E-CPs and Si-CL-CPs

Reusability of Si-E-CPs and Si-CL-CPs was evaluated in repeated cycles with p-nitrophenyl butyrate (p-NPB). The Si-E-CPs and Si-CL-CPs were recovered from the reaction medium by centrifugation at 15,000 rpm for 1 min and washed with tris-HCl buffer (0.1 M, pH 7.2) to remove any residual substrate. The process was repeated for up to seven cycles to examine the reusability of the catalysts. 

### 3.6. Enzymatic Hydrolysis for p-Nitrophenyl Butyrate

Enzymatic hydrolysis was achieved against p-nitrophenyl butyrate (p-NPB) with native CalB, Si-E-CPs, and Si-CL-CPs, respectively. The stock solution of p-NPB (0.1 M) was prepared and diluted in different concentrations (0.125–0.5 mM) with tris-HCl buffer (0.1 M, pH 7.2). Next, 1.3 μL of native CalB (28.3 mg/mL) and 0.1 mg of Si-E-CPs, and Si-CL-CPs were added in 1 mL of substrate solution and incubated for 5 min at R.T. The mixture was centrifuged at 15,000 rpm for 1 min. The absorbance of the product, p-nitrophenol (p-NP), was measured at 400 nm by UV-Vis. The catalytic parameters such as Michaelis–Menten constant (K_m_), maximum reaction velocity (V_max_) and turnover value (K_cat_) were calculated for native CalB, Si-E-CPs, and Si-CL-CPs, respectively.

### 3.7. Enzymatic Synthesis for Benzyl Benzoate with Native CalB and Si-CL-CPs

Benzoic acid and benzyl alcohol were mixed in 1:9 molar ratio for enzymatic esterification. Next, 0.5 g of Si-CL-CPs and 0.185 g of native CalB were added to the mixture and incubated on a rotary shaker at 50 °C for 4, 8, 12, 16, 20, and 24 h, respectively. After the reaction was completed, the supernatant of benzoic acid was separated by centrifugation at 4000 rpm for 20 min. For the enzymatic acylation, benzoic anhydride and benzyl alcohol were mixed in a 1:9 molar ratio. The experiment was carried out in duplicate. The extracted supernatants were separated by column chromatography, and the solvent was evaporated using a rotary evaporator after thin layer chromatography (TLC) analysis. The formation of benzyl benzoate was confirmed by measuring the absorbance at 229 nm using a UV-Vis. spectrophotometer.

### 3.8. Instrumental Analysis

The morphological details of Si-E-CPs and Si-CL-CPs as a function of TEOS concentration were investigated by field emission scanning electronic microscopy (FE-SEM) using a Mira-3 instrument (Tescan, Brno, Czech Republic) with 2 kV accelerating voltage. To evaluate the amount of encapsulated CalB enzyme in Si-E-CPs and Si-CL-CPs, a thermogravimetric analysis (TGA) was performed from room temperature to 700 °C at a heating rate of 10 °C min^−1^ in a nitrogen atmosphere using a Q600 TA instrument (Waters, New Castle, DE, USA). Fourier transform infrared (FT-IR) spectroscopy and dynamic light scattering (DLS) were carried out for identification and characterization of Si-E-CPs and Si-CL-CPs. FT-IR spectra were measured with a KBr compressed pellet in wavelength range of 450–4000 cm^−1^ using an SDT Q600 Frontier (Perkin Elmer, Houston, TX, USA). Particle sizes of Si-E-CPs, and Si-CL-CPs were evaluated using a ZEN 3600 (Malvern, UK). UV-Vis. spectra were measured by Mega 900 (SCINCO, Seoul, Korea) in a range of 190–500 nm. ^1^H-NMR spectra were measured on a Bruker Advance III 400 spectrometer (400 MHz) (Seoul, Korea) using CDCl_3_ solutions and TMS as an internal standard. Chemical shifts are reported in parts per million (ppm, d) relative to the internal tetramethylsilane standard (TMS, d 0.00). The peak patterns are indicated (s, singlet; d, doublet; t, triplet; m, multiplet). Column chromatography was generally performed on silica gel (pore size 60 Å, 32–63 nm particle size) and reactions were monitored by a thin-layer chromatography (TLC) analysis performed with Merck Kieselgel 60 F254 plates and visualized using UV light at 240 nm.

## 4. Conclusions

Si-E-CPs and Si-CL-CPs were prepared as a function of TEOS concentration, in the ranges of 10 to 50 mM and 100 to 300 mM, respectively. The prepared Si-E-CPs and Si-CL-CPs had different particle sizes and morphology, depending on the TEOS concentration, such as uniformed spheres and irregular aggregates. These materials demonstrated thermal and pH stability under various temperature and pH conditions. Reusability was also demonstrated, compared with the native CalB enzyme. With these materials, enzymatic hydrolysis against the p-NPB and enzymatic esterification of benzyl benzoate with one acyl donor in benzoic acid and two acyl donors in benzoic anhydride was demonstrated. The esterification efficiency of Si-CL-CPs was higher in benzoic anhydride than in benzoic acid. Furthermore, the conversion efficiency to benzyl benzoate, corresponding to native CalB and Si-CL-CPs, was 75% and 67%, respectively. Although the conversion efficiency of Si-CL-CPs was not much higher than that of native CalB, it has an efficiency of 91% compared to native CalB, and is expected to be very useful because it has high thermal and pH stability and excellent reusability.

## Figures and Tables

**Table 1:** Michaelis–Menten parameters of native CalB, Si-E-CPs, and Si-CL-CPs as a function of TEOS concentration for hydrolysis of p-NPB.

	[TEOS] (mM)	Km (μM)	Vmax (μM ⸱ min^−^^1^)	Kcat (min^−^^1^)	Kcat/Km (mM^−^^1^ ⸱ min^−^^1^)
Native CalB	0	432.60	16.86	15.03	34.76
Si−E−CPs	10	915.32	4.57	5.03	5.50
	50	588.31	9.94	9.90	16.83
Si−CL−CPs	100	517.31	12.02	10.70	20.68
	300	497.86	12.06	10.73	21.56
